# Supplementary material for: Exogenous Glycine Betaine Application Improves Freezing Tolerance of Cabbage (Brassica oleracea L.) Leaves
Source: Plants (Basel). 2021 Dec 20;10(12):2821. doi: 10.3390/plants10122821 (PMC8703899; doi:10.3390/plants10122821)
Supplement: Supplementary file 1 [file plants-10-02821-s001.zip › plants-1451053-supplementary.pdf]

Preliminary experiment:
The effect of glycine betaine (GB) on freezing tolerance of cabbage (*Brassica oleracea* L. cv. Myeong-Sung) leaves

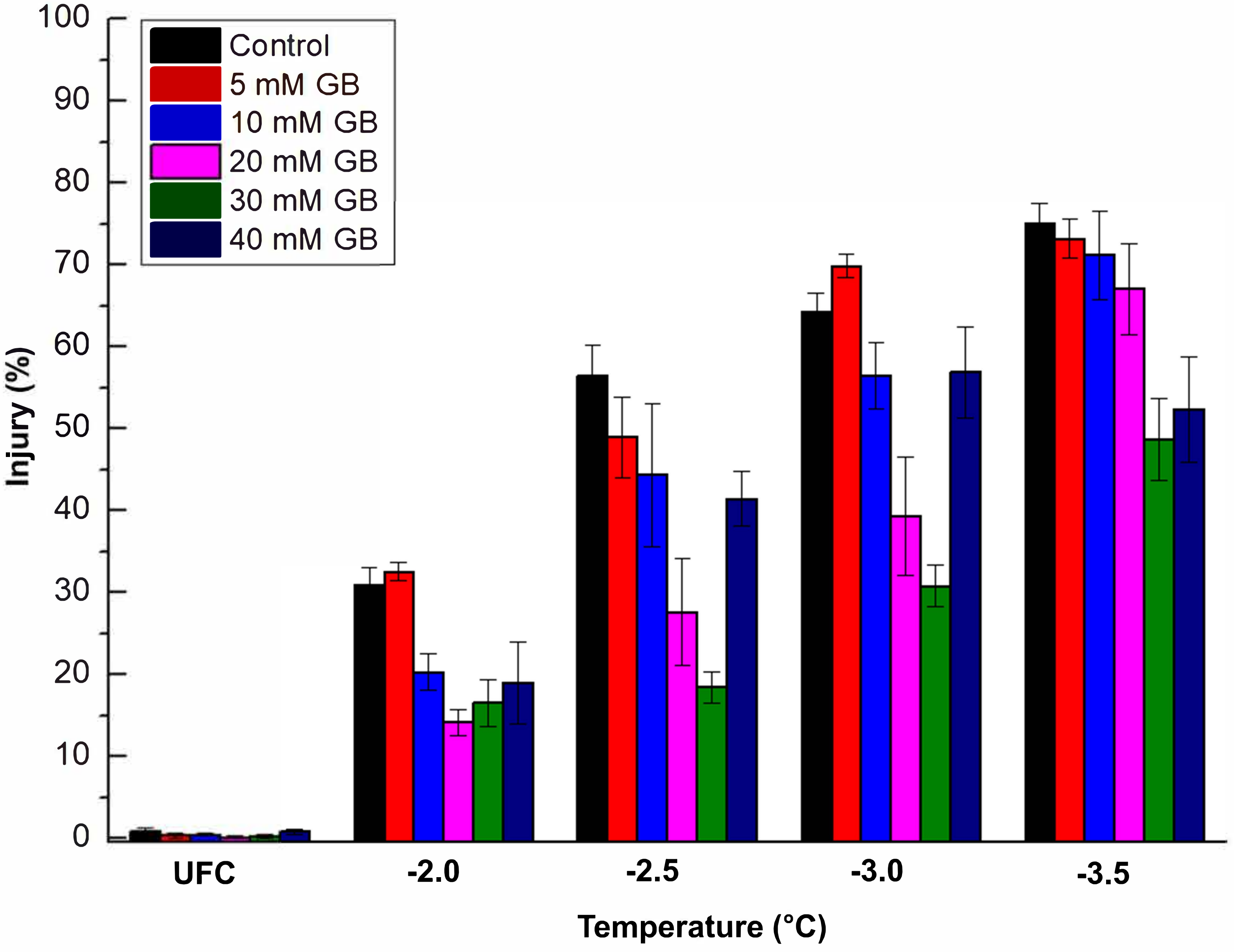

Supplementary Figure S1. Effect of exogenous GB on leaf freezing tolerance of 3-week-old cabbage (*Brassica oleracea* L. cv. Myeong-Sung) seedlings sub-irrigated with water only (control) and water + 5/10/20/30/40 mM GB; injury percent (means ± S.E.) assessed by electrolyte leakage from excised-leaves subjected to freeze-thaw stress at -2.0, -2.5, -3.0, and -3.5 °C. UFC, unfrozen control.
